# ESM1 mediates NGFR-induced invasion and metastasis in murine oral squamous cell carcinoma

**DOI:** 10.18632/oncotarget.12210

**Published:** 2016-09-23

**Authors:** Chen Chen, June Ho Shin, Joshua T. Eggold, Man Ki Chung, Luhua H. Zhang, Jeremy Lee, John B. Sunwoo

**Affiliations:** ^1^ Division of Head and Neck Surgery, Department of Otolaryngology, Stanford University School of Medicine, Stanford, CA 94305, USA; ^2^ Stanford Cancer Institute, Stanford University School of Medicine, Stanford, CA 94305, USA; ^3^ Graduate Program in Cancer Biology, Stanford University School of Medicine, Stanford, CA 94305, USA; ^4^ Department of Otolaryngology Head and Neck Surgery, Shandong Provincial Hospital Affiliated to Shandong University, Jinan, 250021, P.R. China; ^5^ Department of Otorhinolaryngology, Head & Neck Surgery, Sungkyunkwan University School of Medicine, Samsung Medical Center, Sungkyunkwan, Korea

**Keywords:** nerve growth factor receptor, CD271, HNSCC, metastasis, endocan

## Abstract

Oral squamous cell carcinoma (OSCC) is a highly invasive and metastatic malignancy. The nerve growth factor receptor (NGFR) has been observed to be expressed on a subset of cells in OSCC, and NGFR^+^ cells have greater tumor-initiating capacity *in vivo*. Further, inhibition of NGFR reduces tumor growth, indicating a functional role of this receptor; however, the mechanisms by which NGFR confers enhanced tumor formation are not known. Here, we used an established murine model of OSCC and gene expression array analysis to identify *ESM1* as a downstream target gene of NGFR, critical for tumor invasion and metastasis. *ESM1* encodes a protein called endocan, which has the property of regulating proliferation, differentiation, migration, and adhesion of different cell types. Incubation of NGFR^+^ murine OSCC cells with nerve growth factor resulted in increased expression of ESM1. Importantly, *ESM1* overexpression conferred an enhanced migratory, invasive, and metastatic phenotype, similar to what has been correlated with NGFR expression. Conversely, shRNA knockdown of *ESM1* in NGFR overexpressing OSCC cells abrogated the tumor growth kinetics and the invasive and metastatic properties associated with NGFR. Together, our data indicate that NGFR plays an important role in the pathogenesis and progression of OSCC via regulation of *ESM1*.

## INTRODUCTION

Oral squamous cell carcinoma (OSCC) accounts for more than 90% of all malignant lesions of the mouth [1]. Despite advances in therapy, survival rates have not dramatically improved [2]. A series of molecular drivers contribute not only to the initiation of this malignancy but also to its invasive and metastatic properties [3]. In normal oral mucosa epithelium, a sub population of basal cells with stem cell-like properties has been shown to express the nerve growth factor receptor (NGFR), and recent reports indicate that NGFR contributes to the tumor-initiating capacity of a number of malignancies [4, 5]. We have previously shown that NGFR^+^ cells in human OSCC possess the greatest tumor-initiating capacity in this malignancy and that inhibition of NGFR has profound negative effects on the ability of these tumor-initiating cells (TIC) to form tumors *in vivo* [4].

NGFR, also known as p75 neurotrophin receptor (p75NTR) and CD271, is a cell surface receptor that belongs to the tumor necrosis factor receptor superfamily. There are two general classes of neurotrophin receptors: the high-affinity nerve growth factor tyrosine kinase receptors Trk A, B and C (encoded by *NTRK1*, *NTRK2*, and *NTRK3*, respectively) and the low-affinity nerve growth factor receptor NGFR. TrkA binds NGF, while TrkB binds BDNF and NT4, and TrkC binds NT3. Activation of TrkA can inhibit angiogenesis, induce differentiation and growth arrest and mediate apoptosis [6, 7]. In contrast, high intratumoral expression of TrkB and its specific ligand, BDNF, enhances proliferation, metastatic behavior and chemoresistance in neuroblastoma cells [8]. Activation of NGFR results in activation of the NF-κB (nuclear factor-κB) [9, 10], Jun kinase [11, 12] and other signaling pathways. Dependent on the cell type, cell differentiation status, neurotrophin binding, availability of intracellular adaptor molecules, and interacting transmembrane co-receptors and post-translational modification expression, NGFR signal transduction pathways are extremely variable [13]. These variable pathways lead to different cellular responses, such as cell survival [14], apoptosis [14, 15], neurite outgrowth and retraction [16], myelination [17], cell cycle regulation [18], cell migration and invasion [19, 20], and progenitor differentiation [5]. As mentioned, NGFR is expressed not only in nervous tissue, but also in non-neuronal normal and cancer cells, such as head and neck squamous cell carcinoma [4] and breast cancer [21], where it enhances proliferation and promotes cancer metastasis. The precise function of NGFR in OSCC remains unclear. Here, we investigated the mechanism by which NGFR affects invasion and tumor progression in a murine oral squamous cell carcinoma (MOC) model and identified an important downstream target of NGFR to be a gene called *ESM1*.

## RESULTS

### NGFR expression correlates with tumor growth kinetics and invasion in a murine model of oral squamous cell carcinoma

**Figure 1:**
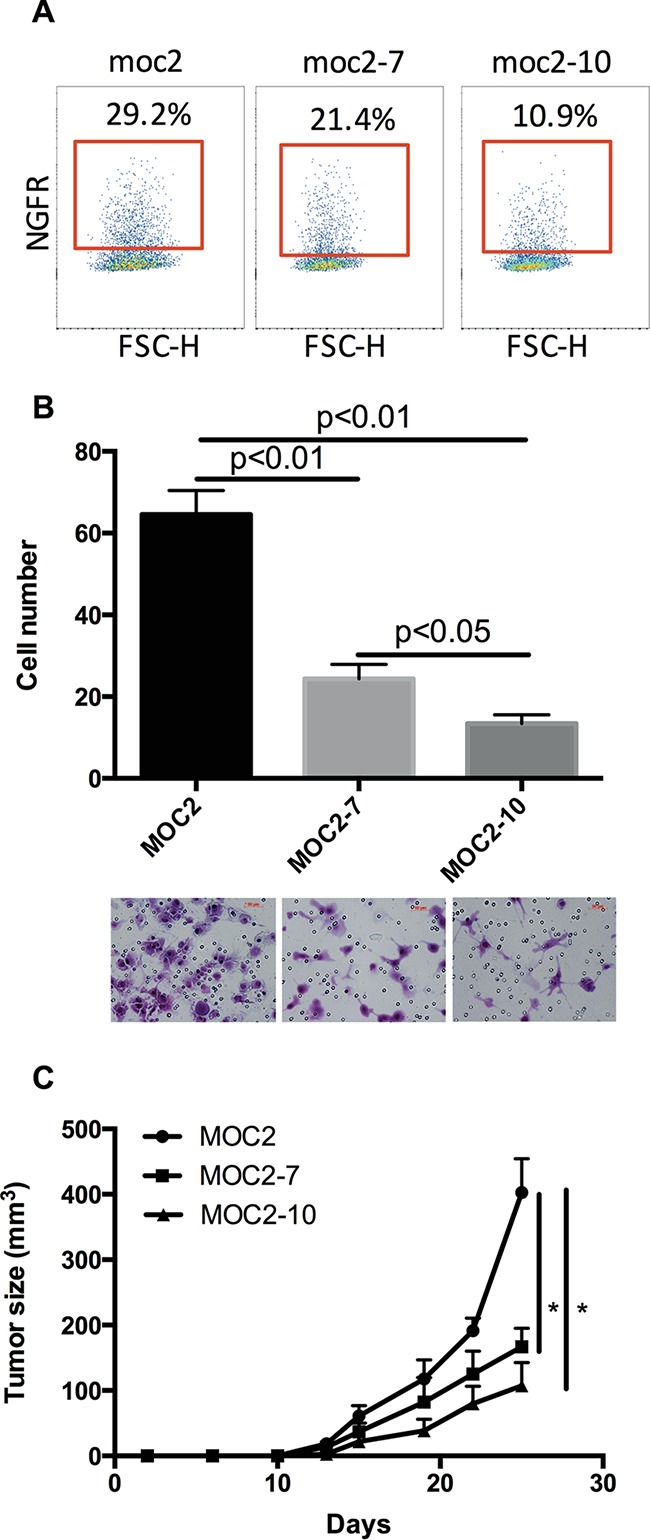
NGFR expression correlates with tumor growth kinetics and invasion in a murine model of oral squamous cell carcinoma **A.** NGFR surface protein expression on MOC2, MOC2-7 and MOC2-10 cells, assessed by flow cytometry, gated on DAPI^−^ cells. **B.** The invasive phenotype of MOC2, MOC2-7 and MOC2-10 cell lines was evaluated by transwell assay *in vitro*. Representative images of crystal violet-stained invasive cells after incubation. Data represent the mean±SEM. **C.** Tumor growth kinetics were assessed after subcutaneous injection with 1×10^4^ cells/mouse of MOC2, MOC2-7 and MOC2-10 in B10; B6-Rag2^−/−^II2rg^−/−^ mice. Each cohort consisted of 5 mice. Data represent the mean±SEM, *p<0.05.

Expression of NGFR in human OSCC is heterogeneous [4]. Examination of three murine OSCC cell lines (MOC2, MOC2-7 and MOC2-10), derived from oral squamous cell carcinoma tumors arising from topical administration of 7,12-dimethylbenz(a)anthracene (DMBA) in the oral cavities of mice [22], also revealed heterogeneous expression of NGFR (Figure 1A). Consistent with previous observations that NGFR contributes to tumor-initiating capacity [4], the degree to which NGFR was expressed in these cell lines appeared to correlate with both tumor cell invasion and migration capacity in transwell invasion assay and tumor growth kinetics *in vivo* (Figure 1B and 1C).

**Figure 2:**
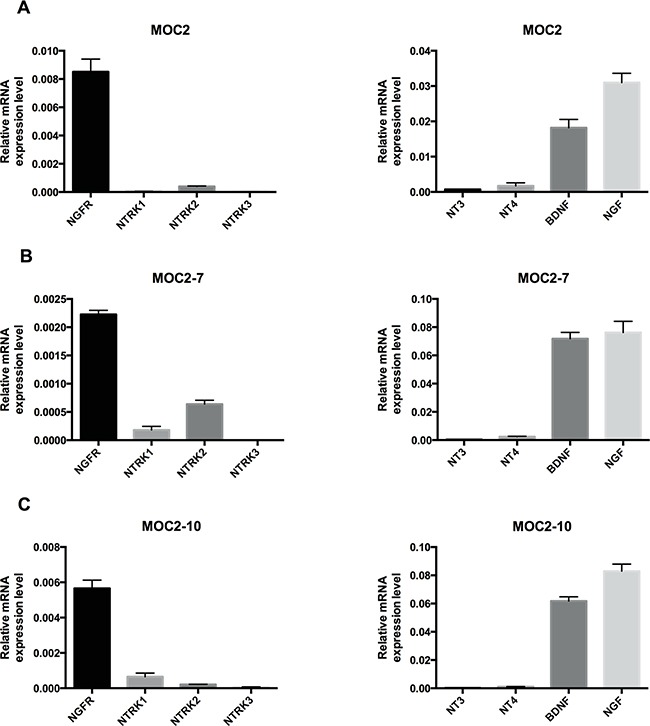
Neurotrophin receptor and neurotrophin expression in murine oral squamous cell carcinoma cell lines Quantitative RT-PCR analysis of *NGFR*, *NTRK1*, *NTRK2*, *NTRK3*, *NT3*, *NT4*, *BDNF* and *NGF* in murine OSCC cell lines: MOC2 **A.** MOC2-7 **B.** and MOC2-10 **C.** Results are presented as units defined as the n-fold difference relative to the control gene *HPRT1*. Data represent the mean±SEM.

To understand the role of NGFR in tumorigenicity, we assessed NGFR expression and related neurotrophin receptors, as well as neurotrophin ligands that bind these receptors, in the murine OSCC cell lines and found that NGFR is expressed in all three cell lines (Figure 2). The expression of other neurotrophin receptors (NTRK1, NTRK2 and NTRK3) was very low relative to NGFR. Since cells often co-express both neurotrophin receptors and cognate neurotrophin ligands (NGF, BDNF, NT-3 and NT-4) to create an autocrine loop, we examined the expression of these neurotrophins in the MOC cell lines. Analysis of transcripts showed that NGF and BDNF are expressed in all three cell lines (Figure 2). These data suggest a role for NGFR signaling in the pathogenic properties of MOC cells.

### NGFR activation upregulates ESM1 expression in MOC cells

**Figure 3:**
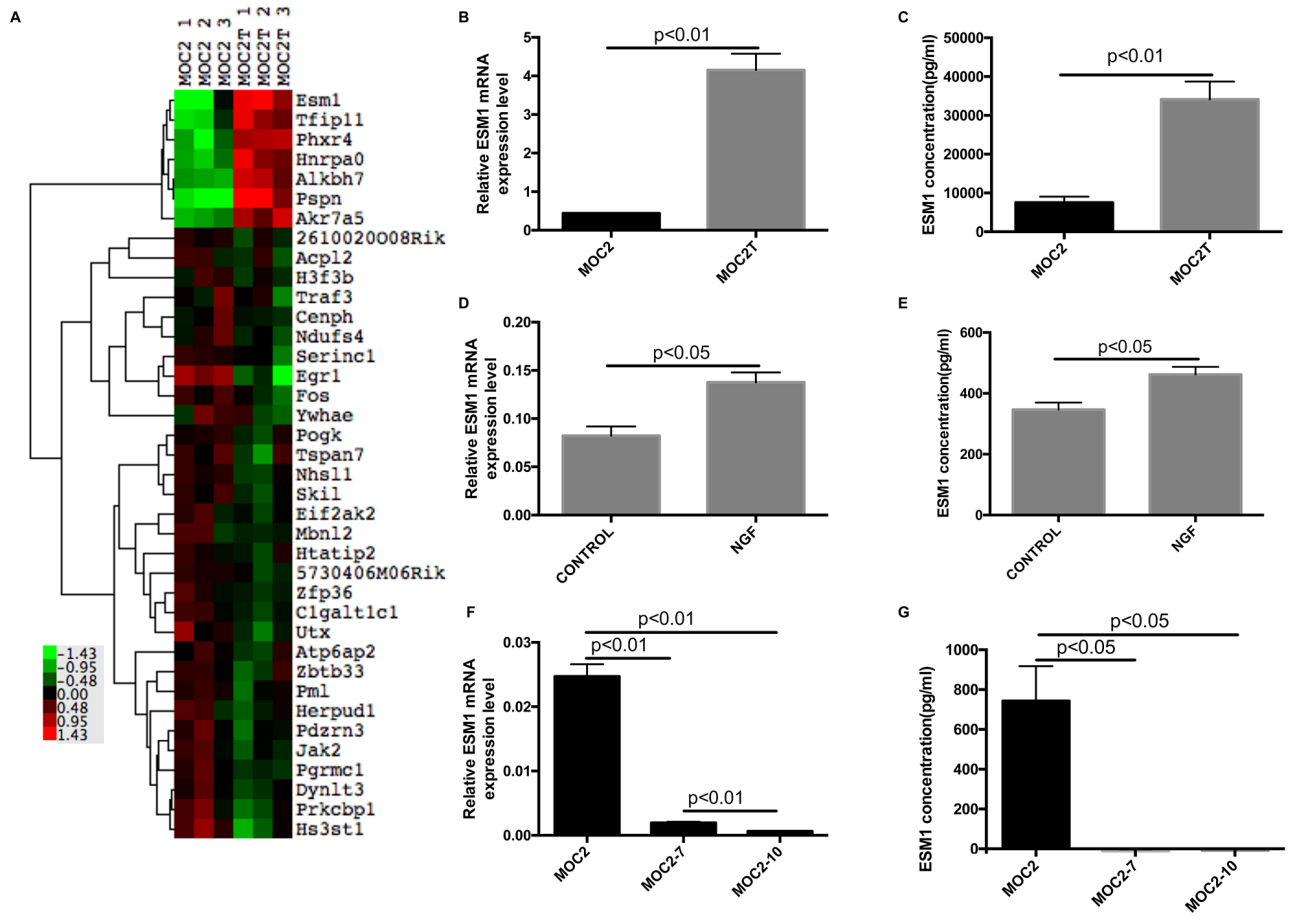
NGFR regulates expression of *ESM1* **A.** Heat map of hierarchical clustering analysis of differentially expressed genes in MOC2 cells and MOC2 cells that overexpress NGFR (MOC2T), selected at p≤0.05 followed by 2-fold cutoff change. Each column represents data from a single cohort with shades of red and green indicating up- or down- regulated genes according to the color scheme shown beside. **B, C.** *ESM1* mRNA expression, assessed by qRT-PCR, and ESM1 soluble protein expression, assessed by ELISA, in MOC2 and MOC2T cells. Data represent the mean±SEM. **D, E.** *ESM1* mRNA expression, assessed by qRT-PCR, and ESM1 soluble protein expression, assessed by ELISA, in MOC2 cells that were incubated *in vitro* with or without 100 ng/ml recombinant human NGF for 24 hours. Data represent the mean±SEM. **F, G.** Transcriptional expression of *ESM1* mRNA, assessed by qRT-PCR, and ESM1 soluble protein expression, assessed by ELISA, in mouse oral squamous cell lines-MOC2, MOC2-7 and MOC2-10. Data represent the mean±SEM. The qRT-PCR results are presented as units defined as the n-fold difference relative to the control gene *HPRT1*.

To explore the potential function of NGFR in MOC cells, we overexpressed NGFR in MOC2 cells and compared the gene expression profiles between MOC2 and an NGFR overexpressing clone of MOC2 (MOC2T) by microarray analysis. Among 45,281 probes analyzed, 38 genes were differentially expressed by at least a two-fold difference (Figure 3A). Of these, 7 were up-regulated, and the rest were down-regulated in MOC2T cells. *ESM1*, one of the most up-regulated genes in the NGFR overexpressing MOC2T cells, was of particular interest given its known role in cancer progression [23]. The *ESM1* differential expression, which was observed with the gene microarray, was confirmed in these cells by qRT-PCR (Figure 3B) and ELISA (Figure 3C).

To assess the effect of NGFR on *ESM1* expression, MOC2 cells were cultured with recombinant human NGF for 24 hours. A significant increase in the expression of *ESM1* was observed with NGF treatment, indicating that NGFR signaling was contributing to the expression of *ESM1* in MOC2 (Figure 3D-3E). Further, comparison of *ESM1* expression in MOC2, MOC2-7, and MOC2-10 cells revealed a correlation with the extent of NGFR expression and the tumor growth kinetics and invasive phenotype observed in the MOC cell lines (Figure 3F-3G and Figure 1). Among the three cell lines, *ESM1* was most highly expressed in MOC2 and least in MOC2-10. Correspondingly, MOC2 was also the most invasive cell line, as measured by *in vitro* transwell invasion assay, and MOC2-10 the least invasive (Figure 1). Since *ESM1* has been shown to contribute to tumor progression in multiple tumor types [24–26], these data suggested that *ESM1* expression may also have a functional role in oral squamous cell carcinoma.

### *ESM1* modulates the invasive phenotype of MOC cells

**Figure 4:**
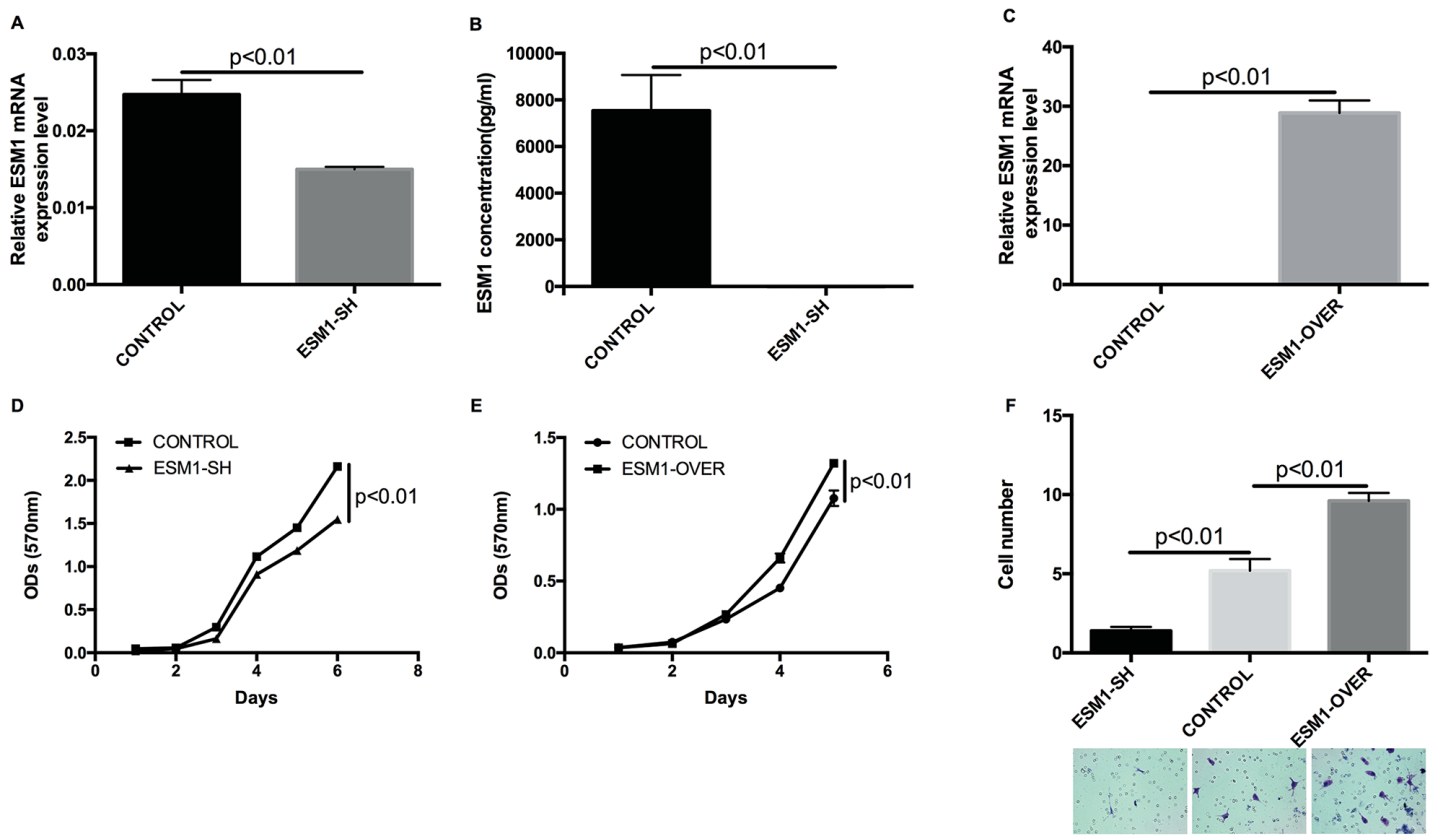
*ESM1* modulates the invasive phenotype in MOC cells **A, B.** *ESM1* mRNA expression, measured by qRT-PCR, and ESM1 soluble protein expression, measured by ELISA, in MOC2 cells after knockdown by *ESM1* shRNA lentiviral transduction. *ESM1* mRNA expression is normalized to *HPRT1* expression. **C.** *ESM1* mRNA expression in MOC2 cells after overexpression by *ESM1* cDNA lentiviral transduction. *ESM1* expression is normalized to *HPRT1* expression. **D.** Cell proliferation/viability of control MOC2 cells and MOC2 cells expressing shRNA targeting *ESM1* (ESM1-SH), measured by MTT assay. **E.** Cell proliferation/viability of control MOC2 cells and MOC2 cells overexpressing *ESM1* (ESM1-OVER), measured by MTT assay. **F.** Cell invasive and migratory capacity, assessed by transwell assay, of MOC2 cells, compared to MOC2 cells either expressing shRNA targeting *ESM1* (ESM1-SH) or overexpressing *ESM1* (ESM1-OVER). Representative images of crystal violet-stained invasive cells after incubation. Data represent the mean±SEM.

To examine the functional role of *ESM1* in MOC cells, shRNA targeting *ESM1* was stably transduced into MOC2 cells (ESM1-SH) to knockdown expression of *ESM1*, and an *ESM1* expression construct was also transduced into MOC2 cell line (ESM1-OVER) to overexpress *ESM1*. *ESM1* knockdown or overexpression was confirmed by qRT-PCR (Figure 4A and 4C). *ESM1* knockdown was also confirmed at the protein level by ELISA (Figure 4B). The effect of *ESM1* expression on cell proliferation/viability was only modest (Figure 4D and 4E); however, there was a profound effect of *ESM1* expression on the invasive phenotype of MOC2. Using transwell chamber assays, we assessed the ability of ESM1-SH and ESM1-OVER for their ability to invade and migrate through a Matrigel matrix. The *ESM1* knockdown MOC2 cells showed a reduction in invasion, compared to the control cells (Figure 4F). Conversely, with the *ESM1* overexpressing MOC2 cells, there was a significant increase in invasion that was observed (Figure 4F). These data indicate that *ESM1* contributes to the invasive phenotype of MOC cells.

### *ESM1* knockdown inhibits MOC tumor growth and metastasis *in vivo*

**Figure 5:**
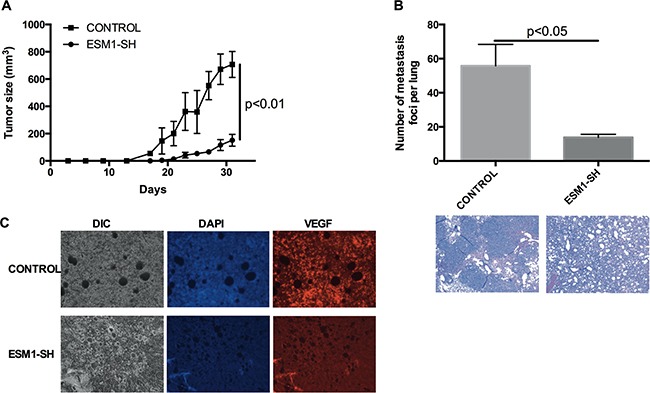
*ESM1* knockdown inhibits MOC tumor growth and metastasis *in vivo* **A.** MOC2 cells and MOC2 cells transduced with *ESM1* shRNA (ESM1-SH) were injected into the subcutaneous compartment over the flanks of mice (3×10^3^ cells/mouse), and subsequent tumor growth was assessed. Each cohort consisted of 5 mice. Data represented the mean±SEM. **B.** The lungs of tumor-bearing mice were assessed for metastatic foci. Data represented the mean±SEM. Representative H&E stains of lungs of the MOC2 control and *ESM1* shRNA knockdown cohorts are shown. **C.** Immunofluorescence imaging of VEGF of representative tumors from the control cohort and the ESM1 shRNA knockdown cohort are shown.

To assess the effect of *ESM1* on tumor growth *in vivo*, MOC2 cells, in which *ESM1* was knocked down (ESM1-SH), and control MOC2 cells were implanted subcutaneously into the flanks of mice, and the growth of the resultant primary tumors was monitored. A significant reduction in tumor growth kinetics and tumor volume was observed in the *ESM1* knockdown cells (Figure 5A). At day 31, the average tumor volume of the control MOC2 cohort was 707.3±78.0 mm^3^, whereas the average tumor volume of the *ESM1* knockdown cohort was 151.6±35.7 mm^3^. These data demonstrated that *ESM1* knockdown effectively suppressed MOC2 tumor growth, despite only modest changes in cell proliferation/viability measured *in vitro* (Figure 4D). Further, there was a profound effect of *ESM1* knockdown on metastasis (Figure 5B). The number of pulmonary metastases resulting from MOC2 primary tumors was significantly higher than that induced by *ESM1* knockdown MOC2 cells (p<0.05). Histologic examination of lung sections confirmed that the pulmonary nodules were invasive squamous cell carcinoma. To assess whether *ESM1* may participate in angiogenesis, VEGF expression was assessed in the tumor sections (Figure 5C), and VEGF expression was observed to be reduced in the *ESM1* knockdown MOC2 tumors. Collectively, these data indicate that *ESM1* contributes to the tumorigenicity, angiogenesis, invasiveness, and metastatic capacity of MOC cells.

### *ESM1* knockdown abrogates the invasive and metastatic phenotype induced by NGFR in MOC cells

**Figure 6:**
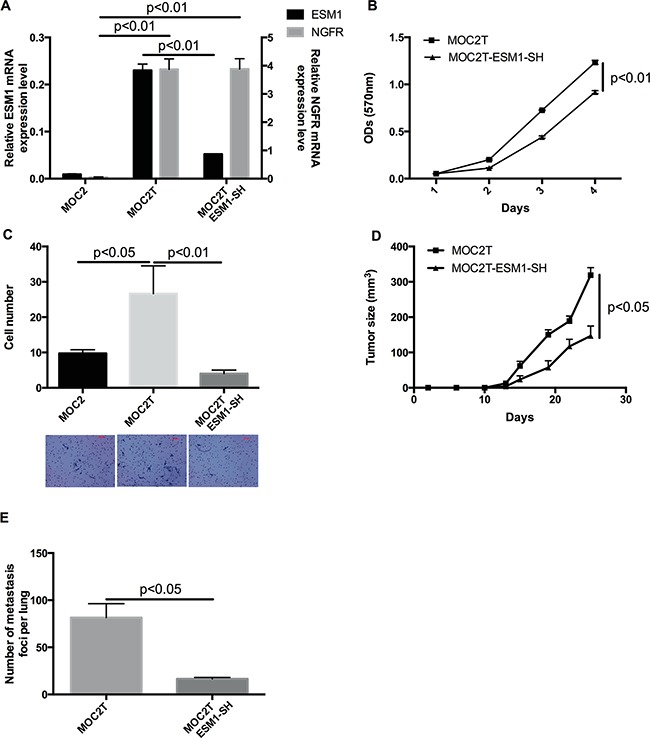
*ESM1* knockdown abrogates the invasive and metastatic phenotype induced by NGFR in MOC cells **A.** Expression of *ESM1* and *NGFR* in MOC2, MOC2T (which overexpress *NGFR*) and MOC2T-ESM1-SH cells (which express an shRNA targeting *ESM1* in the MOC2T cells) measured by qRT-PCR. Data represent the mean±SEM. Results are presented as units defined as the n-fold difference relative to the control gene *HPRT1*. **B.** Cell proliferation/viability of MOC2T and MOC2T-ESM1-SH cells as measured by MTT assay. Data represent the mean±SEM. **C.** Cell invasive and migratory capacity of MOC2, MOC2T, and MOC2T-ESM1-SH cells, measured by transwell invasion/migration assays. Representative images of crystal violet-stained cells after incubation. Data represent the mean±SEM. **D.** Tumor growth after MOC2T and MOC2T-ESM1-SH cells were injected (1×10^4^ cells/mouse) subcutaneously into the flanks of mice. The MOC2T cohort consists of 7 mice and MOC2T-ESM1-SH cohort consists of 8 mice. (p<0.05). Data represented the mean±SEM. **E.** The number of metastatic foci counted on the surface of the lungs harvested from tumor-bearing mice. Data represented the mean±SEM.

To investigate the functional relationship between *ESM1* and NGFR, *ESM1* shRNA was transduced into MOC2T cells (which overexpress NGFR), and the *ESM1* knockdown clones (termed MOC2T-ESM1-SH) were selected by both puromycin resistance and GFP positive sorting. *ESM1* and NGFR mRNA expression levels in MOC2T-ESM1-SH were examined and confirmed by qRT-PCR (Figure 6A). Knockdown of *ESM1* resulted in a modest decrease in cell proliferation/viability compared to parental MOC2T cells. However, *ESM1* knockdown had a significant negative effect on the invasive/migratory capacity of MOC2T cells (Figure 6C). These data indicated that the NGFR-enhanced migratory and invasive capacity of MOC2 cells is dependent on *ESM1* expression.

To determine whether *ESM1* plays an important role in the tumorigenicity and metastasis of MOC2T cells *in vivo*, we injected MOC2T and MOC2T-ESM1-SH cells subcutaneously into mice. Consistent with the effect of altered *ESM1* expression on migration and invasion of MOC2T cells *in vitro*, knockdown of *ESM1* significantly inhibited tumor growth (Figure 6D) and reduced the number of lung metastases (Figure 6E) associated with MOC2T cells *in vivo*. These data confirmed that the NGFR-enhanced migratory and invasive capacity of MOC2 cells is dependent on *ESM1* expression.

## DISCUSSION

Cells with tumor-initiating capacity have the ability to propagate tumor formation *in vivo,* and it has been proposed that these resilient cells contribute to tumor recurrence and poor clinical outcome, given their associated intrinsic metastatic capacity and resistance to chemotherapy and radiation [27–31]. NGFR has been identified as a marker of cells with these properties in multiple malignancies, including head and neck squamous cell carcinoma [4, 32]. Here, we report the identification of a novel downstream target gene of NGFR, *ESM1*. In cell culture, *ESM1* overexpression in murine oral squamous cell carcinoma cells enhanced invasion and migration, whereas *ESM1* knockdown resulted in reduced invasive and migratory capacity. To a limited extent, proliferation of the cells was also affected by altering ESM1 expression; however, the effects on proliferation were much more modest. *In vivo* transplantation of *ESM1*-knockdown cells led to a significant reduction in NGFR-induced tumor growth and pulmonary metastases. Collectively, our data demonstrate that the NGFR-enhanced invasive and migratory capacity of MOC2 cells is dependent on *ESM1* expression.

*ESM1*, which encodes for a protein called endocan, has been previously characterized as an endothelium derived soluble dermatan sulfate proteoglycan (DSPG). It was cloned from a human umbilical vein endothelial cell (HUVEC) cDNA library. *ESM1* is mainly expressed in the vascular endothelium and constitutively circulates in the bloodstream of healthy subjects. It has been found to be dramatically increased in the context of inflammation and cancer, including non-small cell lung cancer, renal clear cell carcinoma, and colorectal cancer etc. [24–26, 33–42]. As a biomarker of neovascularization, *ESM1* overexpression is also often used as an indicator of tumor progression and metastasis in certain malignances, such as glioblastoma, renal cell carcinoma, and non-small cell lung cancer [24–26, 36]. Further, *ESM1* has been found to be strongly associated with tumor invasion in pituitary adenomas [34, 43].

*ESM1* is also known to be overexpressed in tumor cells themselves and can promote tumor growth [23, 44, 45]. *ESM1* interacts with several growth factors, such as hepatocyte growth factor/scatter factor (HGF/SF), by presence of a glycan chain, thereby eliciting epithelial cell migration and growth *in vitro* [46]. In addition, *ESM1* regulates the lymphocyte function-associated antigen-1 (LFA-1)/intercellular adhesion molecule-1 (ICAM-1) pathway; thus, it may play an important role in the migration of leukocytes into tumor tissues [47]. *ESM1* overexpression in non-tumorigenic epithelial cells induces tumor formation in SCID mice [44]. In accordance with these studies, our *in vivo* and *in vitro* data both indicate that altered *ESM1* expression plays an important role in tumor formation and metastasis of murine oral squamous carcinoma. Inhibited expression of *ESM1* in MOC2 was associated with much reduced tumor growth, compared to control cells. These data indicate that *ESM1* may a viable target for antitumor therapy.

Cancer metastasis is a complex process, and its cellular and molecular mechanisms remain unclear. VEGF, a secreted dimeric glycoprotein, is an important regulator of tumor angiogenesis. Previous reports have demonstrate a feedback loop where VEGF-A positively regulates *ESM1* expression, which in turn enhances VEGF-A mediated signaling [38]. Consistent with this, we found that *ESM1* knockdown resulted in significantly lower VEGF expression.

Taken together, our data demonstrate *ESM1* to be a gene target downstream of NGFR and that *ESM1* has an important functional role in the invasive phenotype of murine OSCC. We have demonstrated that *ESM1* knockdown reduces the metastatic capacity of MOC2 cells *in vivo*, providing rationale to investigate the therapeutic potential of targeting NGFR and/or *ESM1* in oral SCC. Current efforts to understand the signaling intermediates between NGFR activation and *ESM1* expression are underway. Finally, *ESM1* expression, alone or in combination with NGFR expression, might serve as novel prognostic biomarker for oral SCC.

## MATERIALS AND METHODS

### Cell lines

The MOC2, MOC2-7, and MOC2-10 murine oral squamous cell carcinoma cell lines were provided by Dr. Ravindra Uppaluri at Washington University in St. Louis, who developed the cell lines from murine oral squamous cell carcinomas induced by topical 7,12-dimethylbenz(a)anthracene (DMBA) administration. Cells were cultured in complete DMEM/F12 medium containing 10% fetal bovine serum (FBS) and 1% penicillin and streptomycin. Cells were maintained at 37°C in a humidified atmosphere containing 5% CO_2_.

### RNA purification and quantitative RT-PCR analysis

Isolated cells were seeded at a density of 1×10^5^ cells/well in a 6-well plate for one day prior to RNA preparation. For NGF-induced gene expression, cells were starved one day in a serum-free medium before treatments with NGF. Then, the cells were treated with or without 100 ng/ml recombinant human NGF (Sigma) in serum-free medium for 24 hours prior to harvest. Total RNA was extracted from the samples using a RNeasy Mini Kit (Qiagen). The cDNA synthesis was performed using a Maxima First Strand cDNA Synthesis Kit (Thermo) following the manufacturer's protocol. The relative amount of gene mRNA was analyzed by quantitative real-time reverse transcription-Polymerase Chain Reaction (qRT-PCR). The qRT-PCR was performed using Luminaris Color Probe High ROX qPCR Master Mix (Thermo). Mouse *HPRT1* was amplified as control. Gene expression was expressed as arbitrary units defined as the n-fold difference relative to the control gene *HPRT1*.

### Enzyme-linked immunosorbent assay

ESM1 protein expression level was measured by enzyme-linked immunosorbent assay (ELISA), using a Mouse ESM1 PicoKine ELISA kit (Boster Biological Technology, Pleasanton, CA). Plates came pre-coated with monoclonal rat anti-ESM1 antibody. Standards and cell culture supernatants were diluted in sample diluent buffer and incubated for 90 minutes at 37°C. Biotinylated polyclonal goat anti-ESM1antibody was diluted in antibody diluent buffer and incubated for 1 hour at 37°C. After washing steps in PBS, Avidin-Biotin-Peroxidase Complex was diluted in ABC diluent buffer and incubated for 45 minutes at 37°C. After washing steps in PBS, TMB color developing agent was added to each well and incubated in the dark for 20 minutes at 37°C. TMB stop solution was added to each well, and absorbance was measured at a wavelength of 450 nm using a microplate (ELISA) reader (SpectraMax M3, Molecular Devices). A six-point standard curve was used to calculate the concentration (pg/mL) of ESM1 in the samples.

### Flow cytometry analysis

Cells were harvested from culture flasks and the single-cell suspensions were incubated with anti-mouse NGFR antibody (mu p75, ATS Bio). DAPI was used to allow exclusion of non-viable cells. NGFR expression was assessed by fluorescence activated cell sorting analysis on a BD LSRFortessa or BD FACSAria II. Events collected were analyzed using FlowJo Version 9.6.4 software (Tree Star).

### Microarray anaylsis

Illumina MouseWG-6 v2 Expression BeadChip was used following the manufacturer's instructions by Genome Technology Access Center in the Department of Genetics at Washington University School of Medicine. We conducted an unpaired student's t-test to compare gene differences between MOC2 and NGFR overexpression MOC2 (MOC2T) cells. Gene expression that showed at least 2-fold increase or decrease and had a significance level of p<0.05 was considered significantly altered after NGFR overexpression.

### Cell proliferation and growth assays

MTT assays were carried out following the manufacturer's instruction (Cell Proliferation Kit I (MTT), Roche). Briefly, cells were seeded into 96-well plates at a concentration of 1,000 cells per well in 100 μl of culture medium. Wells without cells were used as blank controls. Cells were incubated for 1, 2, 3, 4 and 5 days at 37 deg C and 5% CO_2_, and medium was changed every two days. After the incubation period, 10 μl of the MTT labeling reagent was added to each well (final concentration 0.5 mg/ml). After a 4-hour incubation, 100 μl of the solubilization solution was added to each well. Cells were incubated overnight and then tested for complete solubilization of the purple formazan crystals through measuring the absorbance (OD) of the samples at 570nm using a microplate (ELISA) reader (SpectraMax M3, Molecular Devices). All final data were normalized to the OD of the blank controls.

### Lentiviral plasmids

RNAi Consortium (TRC) LentiviralTM shRNA was purchased from GE Healthcare Dharmacon Inc. The shRNA against ESM1 (5′-TCTTTGCATTCCATCCCGAAG-3′) is in pLKO.1-based lentiviral vector. A cDNA encoding *ESM1* from ATG codon to the stop codon of ESM1 was PCR cloned (Forward primer: 5′-GAATTCATGAAGAGCCTCTTGCTGCT-3′; reverse primer: 5′-GGATCCTCAGCGTGGATTTAACCATTTCA-3′) and subcloned BamHI/EcoRI fragments into the pHIV-Zsgreen expression construct, provided by Dr. Michael Clarke (Stanford). This was used for overexpression of *ESM1* in the MOC cells.

### Lentiviral production and transduction

For the production of the lentiviral particles, the HEK 293 cell line was transfected with the packaging plasmid pCMVR8.74, the envelope plasmid pCMV-VSVG and the lentiviral construct containing the shRNA or the transgene, using Lipofectamine® 2000 according to the manufacturer's instructions. Medium was changed 16 hours after the transfection. Virus-containing culture supernatant was collected after 24 hours and centrifuged. Virus was used immediately to infect cells, which were seeded at 3×10^5^ cells per well in a 6-well plate 24 hours prior. Polybrene (8 μg/ml) was also added to enhance the lentiviral transduction efficiency. Medium was changed after 24 hours. In the case of the cells transduced with the pLKO.1 puro vectors, the cell cultures were treated with 1 μg/ml puromycin for one week after media change.

### Invasion and migration assay

To assess the invasive and migratory capacity of the tumor cells, 1×10^4^ cells in 500 μl of serum-free DMEM/F12 was added into the upper chamber and 500 μl of complete medium was placed into the lower chamber (Corning® BioCoatTM Matrigel® Invasion Chamber, Corning). Cells were incubated at 37 deg C for 48 hours, before the non-invading cells were removed from the upper surface of the membrane. After fixation in 95% ethanol for 5 min, the cells still on the opposite surface of the filter membrane were stained with 1% crystal violet for 10 min. The migratory cells were counted in five microscope fields and averaged.

### Animal studies

The B10; B6-Rag2^−/−^II2rg^−/−^ mice (6-11 weeks old, Taconic) were housed in laminar flow cabinets under specific pathogen-free conditions and fed ad libitum. All procedures were performed in accordance with protocols approved by the Administrative Panel on Laboratory Animal Care at Stanford University. Tumor cells were injected subcutaneous in mice. Tumor volume (in mm^3^) was determined by caliper measurements performed every two to three days and calculated by using the following formula: volume=length×width^2^×0.5. The tumors that arose in those mice were harvested when they reached about 1.0 cm in diameter. Lungs were harvested, fixed, and stained with Bouin's solution. Metastatic colonies on the surface of lungs were counted. Tissues were fixed with 10% formalin, and then the paraffin-embedded. H&E stained slides were made by the Department of Comparative Medicine Histology Service Center at Stanford.

### Immunofluorescence

After the formalin-fixed paraffin-embedded specimens were deparaffinized and rehydrated, antigen retrieval was performed. Samples were blocked at 4 deg C overnight in 5% FBS/PBST, and then incubated for 4 hours at room temperature in Anti-VEGF antibody (Novus biologicals) (1:50) diluted with 5% FBS/PBST. Negative control of 5% FBS/PBST was also used.

### Statistical analysis

Data were expressed as mean ±standard error of mean (SEM) and statistically analyzed by t-test. Statistical analysis was performed by IBM SPSS 22 software package. Values of p<0.05 were considered to be statistically significant.
